# Supplementary material for: Mosaic sarbecovirus nanoparticles elicit cross-reactive responses in pre-vaccinated animals
Source: Cell. 2024 Oct 3;187(20):5554–5571.e19. doi: 10.1016/j.cell.2024.07.052 (PMC11460329; doi:10.1016/j.cell.2024.07.052)
Supplement: Table S1. Vaccines and vaccine sources used in immunization experiments, related to Figures 2, 3, 4, 5, and 6 [file mmc1.pdf]

Cell, Volume *187*

Supplemental information

Mosaic sarbecovirus nanoparticles elicit cross-reactive responses in pre-vaccinated animals

Alexander A. Cohen, Jennifer R. Keeffe, Ariën Schiepers, Sandra E. Dross, Allison J. Greaney, Annie V. Rorick, Han Gao, Priyanthi N.P. Gnanapragasam, Chengcheng Fan, Anthony P. West Jr., Arlene I. Ramsingh, Jesse H. Erasmus, Janice D. Pata, Hiromi Muramatsu, Norbert Pardi, Paulo J.C. Lin, Scott Baxter, Rita Cruz, Martina Quintanar-Audelo, Ellis Robb, Cristina Serrano-Amatriain, Leonardo Magneschi, Ian G. Fotheringham, Deborah H. Fuller, Gabriel D. Victora, and Pamela J. Bjorkman

Table S1. Vaccines and vaccine sources used in immunization experiments, related to Figures 2-6.

Animal Study	Vaccinated with		Vaccine Source	Immunized with	Immunogen Source	Figures
pre-vaccinated non-human primates (NHPs)	WA1 Spike, Bivalent (Beta/Delta) SHARP, Trivalent (WA1/Beta/Delta) SHARP	DNA-gene gun	University of Washington, HDT Bio, University of Albany, Creative Biosolutions	mosaic-8b	Caltech	2, S2
		repRNA-LION				
		repRNA-LION				
		repRNA-gene gun				
		repRNA-gene gun				
		DNA-gene gun		homotypic SARS-2	Caltech	
		DNA-gene gun				
		repRNA-LION				
		repRNA-gene gun				
		repRNA-gene gun				
		DNA-gene gun		WA1/BA.1 rep-RNA	HDT Bio	
		DNA-gene gun				
		repRNA-LION				
		repRNA-gene gun				
pre-vaccinated BALB/c mice	Pfizer-like WA1		Helix Biotech	mosaic-8b	Caltech/Ingenza	3, S3
				admix-8b	Caltech/Ingenza	
				homotypic SARS-2	Caltech/Ingenza	
				Pfizer-like WA1 mRNA-LNP	Helix Biotech	
	Pfizer-like WA1 and WA1/BA.5 mRNA-LNP (Bivalent)			mosaic-8b	Caltech/Ingenza	
				mosaic-7	Caltech	
				Pfizer-like WA1/BA.5 mRNA-LNP (Bivalent)	Helix Biotech	
pre-vaccinated BALB/c mice	WA1 ChAdOx1		Jenner Institute, Oxford	mosaic-8b	Caltech	4, 5, S4
				admix-8b	Caltech	
				homotypic SARS-2	Caltech	
				WA1 mRNA-LNP	Rockefeller	
				WA1 ChAdOx1	Jenner Institute, Oxford	
pre-vaccinated S1pr2-IgkTag mice	WA1 mRNA-LNP		University of Pennsylvania, Acuitas	mosaic-8b	Caltech	6, S6
				admix-8b	Caltech	
